# Robotic-assisted total knee replacement: a narrative review of evolution, clinical impact, and future prospects in AI-driven precision surgery

**DOI:** 10.1097/MS9.0000000000004759

**Published:** 2026-03-06

**Authors:** Mohamed Baklola, Naji Al-Bawah, Alaa Jaffar Mohammed, Abdullah Bader Y Aljaffar, Hind Yahya Alyousef, Meshal Saud Alanazi, Amer Ahmed Alotaibi, Yazeed Melwah Alanzi, Reham Nasser Alsaud, Sami Mohammed Alanezi, Abdullah Almotheby

**Affiliations:** a Faculty of Medicine, Mansoura University, Mansoura, Egypt; b Faculty of Medicine, Sana’a University, Sana’a, Yemen; c College of Medicine, Imam Abdulrahman Bin Faisal University, Dammam, Saudi Arabia; d College of Medicine, Al Jouf university, Al Jouf, Saudi Arabia; e College of Medicine, Vision Colleges, Riyadh, Saudi Arabia; f College of Medicine, Umm Al-Qura University, Makkah, Saudi Arabia; g Department of Orthopaedic, Ad Diriyah Hospital, Riyadh, Saudi Arabia

**Keywords:** artificial intelligence, knee arthroplasty, machine learning, robotic-assisted TKR, surgical precision

## Abstract

**Introduction:**

Robotic-assisted total knee replacement (RA-TKR) has established a new standard for surgical precision. However, the translation of this mechanical accuracy into consistently superior long-term clinical outcomes remains debated. This has shifted focus toward the integration of artificial intelligence (AI) and machine learning (ML), not as separate tools, but as synergistic partners to robotics.

**Aim:**

This narrative review proposes a conceptual framework for an AI-driven robotic ecosystem in total knee arthroplasty. We examine the evidence supporting the independent contributions of robotic precision and AI-based analytics and explore how their integration may support a data-informed, adaptive surgical workflow.

**Materials and methods:**

A structured narrative review of peer-reviewed literature on RA-TKR and AI/ML applications in orthopedics was conducted. Evidence was synthesized to support the proposed ecosystem framework.

**Results:**

The evidence confirms that robotic platforms consistently improve implant alignment and reduce outliers, though their impact on long-term patient-reported outcomes is less clear. In parallel, AI/ML applications demonstrate significant capabilities across the surgical workflow, including predictive analytics for preoperative planning, real-time intraoperative guidance, and personalized postoperative outcome forecasting. Our synthesis reveals that the synergy of these technologies creates a feedback loop where surgical and outcomes data continuously refine predictive models.

**Conclusion:**

The future of knee arthroplasty is likely to depend not solely on enhanced mechanical precision but on the judicious integration of robotics with data-driven intelligence. An AI-driven robotic ecosystem offers a promising conceptual model for advancing personalized, predictive care; however, its clinical value and economic sustainability require validation through robust prospective studies.

## Introduction

Total knee arthroplasty (TKA) is a highly successful procedure for treating end-stage knee osteoarthritis, effectively reducing pain and restoring function for patients^[^1^]^. However, the long-term success of conventional TKA (cTKA) is highly dependent on achieving precise implant alignment and optimal soft tissue balancing, outcomes that remain technically demanding^[^2^]^. Inaccuracies in component positioning are associated with persistent pain, instability, and a higher risk of aseptic loosening, potentially leading to premature revision surgery^[^3^]^.

To address these challenges, robotic-assisted TKA (RA-TKA) has emerged as a significant technological advancement^[^4^]^. Modern robotic platforms, including MAKO, ROSA, and VELYS, leverage advanced imaging and intraoperative guidance to enhance surgical precision. Numerous studies have confirmed that RA-TKA consistently improves the accuracy of implant positioning and reduces alignment outliers when compared to manual techniques^[^5–7^]^.

Despite these gains in procedural accuracy, the impact of RA-TKA on long-term functional outcomes, patient satisfaction, and implant survivorship remains a subject of ongoing investigation^[^8^]^. This has led to the proposal that the next paradigm shift in arthroplasty is not merely a more precise robot but a smarter robot powered by the integration of artificial intelligence (AI) and machine learning (ML). This convergence promises to create a data-driven surgical ecosystem capable of optimizing preoperative planning, providing intelligent intraoperative decision support, and personalizing postoperative care.

This review examines the evolution of RA-TKA, critically appraises the current evidence regarding its clinical and functional impact, and evaluates the emerging role of AI and ML as enabling technologies for more intelligent and personalized knee arthroplasty. In addition, we highlight key methodological, technical, economic, and ethical challenges that must be addressed to translate these innovations into validated clinical practice. In accordance with the TITAN Guidelines 2025, we affirm that AI tools were used solely for language refinement and not for data generation, analysis, or conceptual content creation. This declaration is made in compliance with current best practices for the responsible use of AI in medical research and publishing^[^9^]^.

## Methods

### Review design and rational

This study was conducted as a structured narrative review aimed at synthesizing current evidence on RA-TKA and AI/ML applications in orthopedic surgery, with the specific objective of proposing a conceptual framework for an AI-driven robotic ecosystem in knee arthroplasty.

A narrative review methodology was deliberately selected rather than a systematic review or meta-analysis due to the substantial heterogeneity across the available literature. This heterogeneity includes wide variation in robotic platforms, AI methodologies, study designs, outcome measures, follow-up durations, and economic evaluation models. In addition, many AI-focused studies remain exploratory or proof-of-concept in nature and are not amenable to quantitative pooling.

The purpose of this review was therefore not statistical aggregation of outcomes, but rather a qualitative and thematic synthesis that integrates clinical evidence, technical innovation, and health-system considerations. This approach enables critical discussion of emerging synergies between robotics and AI, identification of unresolved challenges, and articulation of future research directions in intelligent knee arthroplasty.

### Search strategy

A comprehensive literature search was conducted across four electronic databases: PubMed (MEDLINE), Scopus, Web of Science, and Google Scholar. All databases were searched from inception until 10 December 2025, which represents the date of the final search.

The search strategy combined Medical Subject Headings and free-text terms related to robotic knee arthroplasty and AI. Core search terms included:

- “robotic-assisted total knee arthroplasty” OR “robotic knee replacement”
- “artificial intelligence” OR “machine learning” OR “deep learning”
- “orthopedic surgery”
- “precision surgery.”

These terms were combined using Boolean operators (AND/OR). A representative PubMed search string was as follows:

(“robotic-assisted total knee arthroplasty” OR “robotic knee replacement”) AND (“artificial intelligence” OR “machine learning” OR “deep learning”) AND (“orthopaedic surgery” OR “precision surgery”).

HIGHLIGHT

- Robotic-assisted total knee replacement (TKR) significantly enhances implant precision and reduces alignment outliers
- Artificial intelligence (AI) transforms TKR from mechanical precision to data-driven intelligence, optimizing planning, intraoperative guidance, and postoperative prediction
- The AI-driven robotic ecosystem creates a continuous feedback loop
- This synergistic ecosystem promises to personalize care and democratize surgical expertise
- Overcoming challenges in data integrity, ethical accountability, and economic viability is crucial for the widespread adoption of this integrated AI-robotics paradigm

Equivalent syntax was adapted for the remaining databases. The complete search strategy for all databases is provided in Supplemental Digital Content File S1, available at: http://links.lww.com/MS9/B96.

To ensure comprehensive coverage, the electronic search was supplemented by a manual search that included:

- Screening reference lists of all included articles,
- Review of relevant systematic reviews and meta-analyses,
- Hand-searching abstracts from major orthopedic and arthroplasty conferences published within the last 5 years.

### Study selection

All retrieved records were imported into EndNote (Clarivate Analytics) for reference management. Duplicate records were identified and removed electronically and through manual verification. Titles and abstracts were then independently screened for relevance, followed by full-text review of potentially eligible studies.

Studies were included if they met the following criteria:

- Reported on RA-TKA and/or applications of AI or ML in orthopedic surgery relevant to knee arthroplasty.
- Provided data on surgical accuracy, clinical outcomes, patient-reported outcome measures (PROMs), complications, healthcare utilization, cost-effectiveness, or future technological directions.
- Were peer-reviewed and published in English.

Exclusion criteria were:

- Studies unrelated to knee arthroplasty or orthopedic applications of AI/ML.
- Non-peer-reviewed literature.
- Case reports or technical notes without evaluative outcomes.
- Non-English publications.

**Figure 1.**
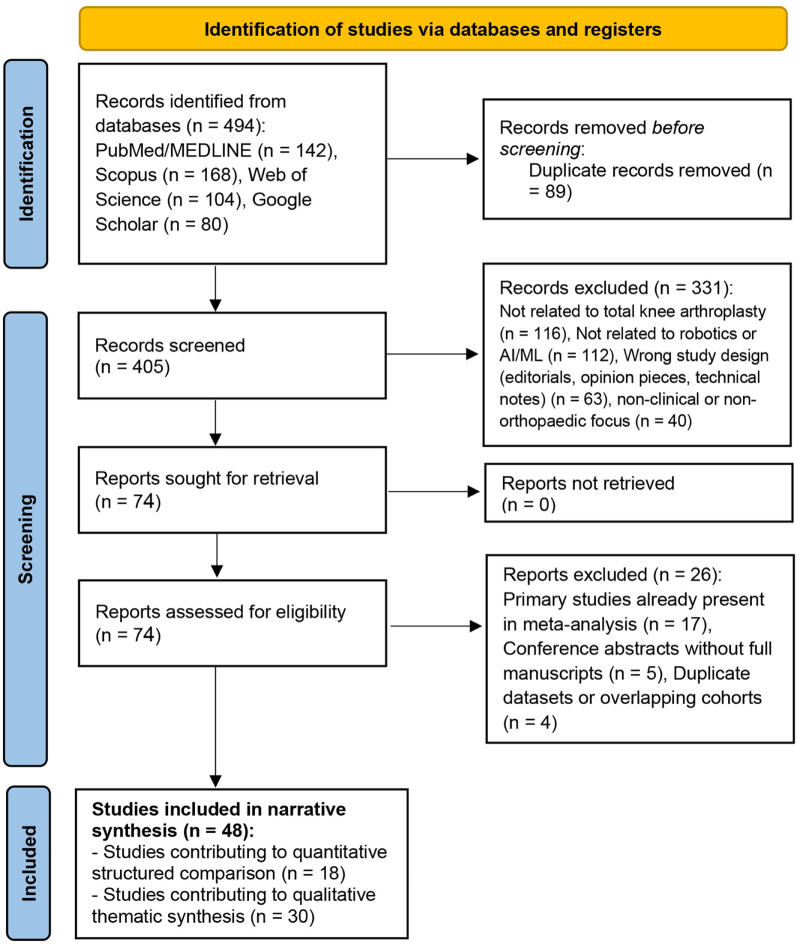
Flow chart of literature identification, screening, eligibility assessment, and inclusion for the narrative review.

The initial database search identified 494 records. After removal of 89 duplicate records, 405 articles underwent title and abstract screening. Of these, 74 reports were assessed at full-text level for eligibility. A flow chart detailing the study identification, screening, eligibility assessment, and inclusion process is presented in Figure 1 to enhance transparency of the literature selection process.

For analytical clarity, included studies were categorized into two groups:

1. Quantitative evidence set (*n* = 18):

Studies reporting numerical comparative outcomes related to RA-TKA performance, clinical results, economic outcomes, or AI/ML model performance metrics (e.g., accuracy and area under the curve (AUC)). These studies informed structured comparison and tabulation.

2. Qualitative evidence set (*n* = 30):

Studies contributing to thematic analysis, conceptual development, technological context, and future perspectives but not suitable for structured quantitative comparison.

Where available, data were preferentially extracted from systematic reviews and meta-analyses rather than individual primary studies. Primary studies were used when higher-level evidence was unavailable or when addressing emerging AI applications.

### Narrative synthesis framework

Data were synthesized using a thematic narrative approach. Evidence was grouped according to:

- Historical evolution of robotic-assisted knee arthroplasty.
- Clinical performance and limitations of current robotic platforms.
- AI/ML applications across the preoperative, intraoperative, and postoperative phases.
- Economic, ethical, and regulatory considerations.
- Integration of robotics and AI into a proposed learning ecosystem.

This framework enabled structured comparison while maintaining flexibility to integrate diverse forms of evidence relevant to the review objectives.

### Critical appraisal and risk of bias considerations

Given the narrative nature of this review, formal quality grading tools such as GRADE, ROBINS-I, or CASP were not systematically applied. However, methodological quality and risk of bias were explicitly considered during evidence interpretation.

Particular attention was paid to:

- Study design (randomized vs observational).
- Sample size and follow-up duration.
- Consistency of outcome measures.
- Presence of industry sponsorship.
- Generalizability of findings across healthcare settings.

Potential sources of bias, including short- to mid-term follow-up, heterogeneity of outcome reporting, and industry involvement, are explicitly discussed in the Limitations section. This critical appraisal informed the cautious interpretation of findings and reinforces that conclusions regarding AI-driven robotic ecosystems remain forward looking and hypothesis generating, rather than definitive.

## Historical development of robotic-assisted TKR

### Evolution of knee arthroplasty techniques

The first knee arthroplasty procedures were performed in the mid-20th century to treat end-stage knee osteoarthritis^[^6^]^. Early prostheses were simple hinged designs that provided limited stability and often restricted, rather than restored, natural knee kinematics^[^10^]^. The 1970s saw the introduction of more technologically complex total knee replacement (TKR) designs; however, many of these early models experienced high failure rates due to biomechanical and material deficiencies^[^11^]^. Consequently, subsequent research efforts focused on creating implants that more closely replicated the knee’s natural anatomy and biomechanics. A pivotal shift occurred in the late 1990s with the introduction of computer-assisted surgery (CAS) into TKR, which laid the groundwork for modern robotics^[^12^]^. CAS systems utilize optical trackers and sophisticated software to provide surgeons with real-time, three-dimensional visualization of the patient’s anatomy. This technology significantly improves the accuracy of bone cuts and component positioning compared to traditional manual instrumentation^[^12^]^.

### Introduction of robotic assistance in knee replacement

The first dedicated robotic system for arthroplasty, the ACROBOT (Active Constraint Robot), was developed at Imperial College London in 1988^[^13^]^. It functioned as an active constraint device, using a robotic arm to precisely guide a cutting tool for bone preparation based on a pre-defined surgical plan. This was followed by the CASPAR system (URS Ortho, Rastatt, Germany), which was developed specifically for TKR and entered clinical use after initial trials in 2000^[^13^]^. Subsequently, the ROBODOC system (now THINK Surgical Inc.) became the first robotic platform to receive FDA clearance for orthopedic surgery, initially for total hip arthroplasty^[^14^]^. Its application was later extended to knee arthroplasty, gaining significant traction in markets such as South Korea, where over 2000 procedures had been performed by 2007^[^15^]^.

### Key technological advancements over time

The evolution of RA-TKA has been driven by the pursuit of greater surgical precision and improved patient outcomes. Key technological advancements include:

1. **Haptic feedback**: Pioneered by systems like the MAKO Robotic-Arm Interactive Orthopedic System, haptic technology provides tactile feedback that constrains the cutting tool within a predefined safety boundary. This helps surgeons execute precise bone resections while minimizing the risk of damage to surrounding soft tissues^[^8^]^.
2. **Image-based and imageless navigation**: Modern systems utilize different navigation strategies. Image-based platforms (e.g., MAKO) rely on preoperative CT scans to create a patient-specific 3D model for surgical planning^[^8^]^. In contrast, imageless systems (e.g., NAVIO) use intraoperative kinematic mapping and landmark registration to build the 3D model in real-time, eliminating the need for preoperative radiation exposure^[^8^]^.
3. **Real-time intraoperative adjustments**: Contemporary platforms like the ROSA Knee System (Zimmer Biomet) provide dynamic, real-time data on limb alignment and soft-tissue tension. This allows surgeons to make intraoperative adjustments to the surgical plan, helping to optimize final implant positioning and achieve ligamentous balance^[^8^]^.

## The foundation: robotic platforms as precision instruments

A comprehensive review of the evidence for major robotic systems, including ROBODOC, NAVIO, MAKO, ROSA, and VELYS, reveals a consistent pattern: while robotic technology is unequivocally successful in achieving its primary goal of precision, its broader impact on long-term functional outcomes and economic viability remains a complex and evolving discussion^[^16,22^]^. The key evidence from recent high-impact meta-analyses and large cohort studies is summarized in Table 1.

**Table 1:** Summary of key comparative studies on robotic assisted vs. conventional TKA.

Robotic system	Study (Year)	Study design	Key findings
ROBODOC/NAVIO	Alrajeb *et al*, 2024^[^16^]^	SR/MA of 7 RCTs (*N* = 1942)	**Accuracy and Alignment**: Improved anatomical alignment and mechanical axis restoration.
			**Clinical Outcomes and Safety**: No significant difference in ROM, PROMs, or complications.
			**Efficiency and Cost**: Not specified.
MAKO (UKA)	Zhang *et al*, 2022^[^17^]^	SR/MA of 14 studies	**Accuracy and Alignment**: Improved precision in implant positioning.
			**Clinical Outcomes and Safety**: Reduced complications. No difference in KSS, WOMAC, or reintervention rates.
			**Efficiency and Cost**: Learning curve of ~6 cases for OR time. Cost-effective only at high surgical volume.
NAVIO (Imageless)	Sun *et al* 2025^[^18^]^	MA of 21 studies (*N* = 2483)	**Accuracy and Alignment**: Improved accuracy; reduced outliers in HKA, FFC, FTC, LDFA, and PTS.
			**Clinical Outcomes and Safety**: No significant difference in PROMs, LOS, or complications. Reduced blood loss.
			**Efficiency and Cost**: Increased operative time.
ROSA	Hax *et al*, 2024^[^20^]^	Registry-based cohort	**Accuracy and Alignment**: Improved coronal plane accuracy and tibial sagittal angle.
			**Clinical Outcomes and Safety**: Improved Oxford Knee Score (OKS) at 6 months. Reduced length of stay.
			**Efficiency and Cost**: Increased operative time and blood loss.
VELYS	Alton *et al*, 2025^[^21^]^	Prospective cohort (*N* = 200)	**Accuracy and Alignment**: Improved femoral/tibial alignment (mMDFA, mMPTA, and TPS).
			**Clinical Outcomes and Safety**: Reduced serious adverse events. Equivalent or improved PROMs at 1 year.
			**Efficiency and Cost**: Not specified.
	Huang *et al*, 2024^[^22^]^	Large database cohort	**Accuracy and Alignment**: Not specified.
			**Clinical Outcomes and Safety**: Reduced 90-day revisits and knee-related readmissions. No difference in revision rates.
			**Efficiency and Cost**: Slightly increased operative time. Similar 90-day costs.

AE, adverse event; cTKA, conventional total knee arthroplasty; FFC, femoral flexion component; FJS-12, Forgotten Joint Score; FTC, femoral tibial component; HKA, hip-knee-ankle angle; KSS, Knee Society Score; LDFA, lateral distal femoral angle; LOS, length of stay; MA, meta-Analysis; mMDFA, mechanical medial distal femoral angle; mMPTA, mechanical medial proximal tibial angle; OKS, Oxford Knee Score; OR, odds ratio; PROMs, patient-reported outcome measures; PTS, posterior tibial slope; RA-TKA, robotic-assisted total knee arthroplasty; RCT, randomized controlled trial; ROM, range of motion; SR, systematic review; TPS, tibial posterior slope; UKA, unicompartmental knee arthroplasty; WOMAC, Western Ontario and McMaster Universities Osteoarthritis Index.

### The consistent advantage: unmatched surgical accuracy

A robust body of literature confirms that the primary strength of RA-TKA lies in its ability to execute a surgical plan with unparalleled accuracy. Multiple systematic reviews and meta-analyses have shown that robotic systems significantly reduce outliers in achieving the desired mechanical axis of the lower limb. For instance, a meta-analysis by Alrajeb *et al* (2024), which synthesized data from seven randomized controlled trials involving the ROBODOC and NAVIO systems, found statistically significant improvements in both anatomical alignment and mechanical axis restoration compared to cTKA^[^16^]^. Similarly, Sun *et al* (2025) analyzed 21 studies on the imageless NAVIO system and reported a significant reduction in outliers across multiple critical alignment parameters, including the hip-knee-ankle angle (HKA), femoral flexion, and posterior tibial slope^[^18^]^.

This trend of superior accuracy is consistent across different platforms. Studies on the ROSA system have demonstrated improved accuracy, particularly in the coronal plane, with tibial sagittal angle alignment being significantly closer to the neutral target^[^20^]^. More recently, the VELYS platform was shown to achieve non-inferiority in HKA restoration while demonstrating significantly higher accuracy in individual femoral and tibial component positioning (mMDFA, mMPTA, and TPS) in a multicenter prospective study^[^21^]^. This collective evidence firmly establishes that robotic platforms function as highly effective precision instruments, minimizing the human error associated with manual jigs and cutting guides and ensuring that the implant is placed according to the preoperative plan.

### The precision–outcome paradox: why improved alignment does not consistently translate into superior patient-reported outcomes

Despite the well-documented improvements in radiographic accuracy achieved with RA-TKA, the translation of these gains into consistently superior PROMs remains inconclusive. Multiple high-quality studies have failed to demonstrate durable, long-term functional advantages of RA-TKA over cTKA. For example, the meta-analysis by Alrajeb *et al* (2024) found no significant differences in postoperative range of motion or global PROMs between robotic and conventional techniques^[^16^]^. Similarly, Sun *et al* (2025) reported no significant advantages of the NAVIO system with respect to pain scores, Knee Society Scores, or Forgotten Joint Scores^[^18^]^. This apparent discordance between radiographic precision and patient-perceived benefit has emerged as a central paradox in contemporary knee arthroplasty.

Several mechanistic explanations may underlie this precision–outcome disconnect. First, it remains uncertain whether traditional mechanical alignment targets represent the optimal biomechanical goal for all patients. Recent meta-analyses comparing kinematic and mechanical alignment strategies in TKA have reported no significant differences in PROMs despite differences in biomechanical targets, suggesting that neutral mechanical axis restoration may not universally translate into better patient outcomes^[^23^]^. Concepts such as kinematic or functional alignment propose that patient-specific alignment strategies may better preserve physiological kinematics, potentially exerting a greater influence on satisfaction than marginal improvements in mechanical accuracy alone^[^23^]^.

Second, soft-tissue balancing may play a more dominant role in determining functional outcomes than incremental improvements in bone resection precision. Although robotic systems enhance precision of component positioning, ligament tension and dynamic joint behavior are critical determinants of stability and comfort that are not fully captured by static alignment metrics^[^24^]^. Third, patient-related factors such as preoperative expectations, pain perception, psychological health, and activity level are known to influence PROMs after TKA. Studies show that patients’ expectations strongly correlate with postoperative satisfaction, independent of radiographic measures of alignment or precision^[^25^]^. Furthermore, some clinical studies report early functional advantages with alignment techniques that respect native joint motion patterns, consistent with the idea that patient-specific biomechanics and recovery experience contribute substantially to outcomes^[^26^]^.

Nevertheless, some evidence suggests that robotic assistance may confer short-term or perioperative advantages. Hax *et al* (2024) observed significantly higher Oxford Knee Scores at 6 months in patients undergoing RA-TKA compared with cTKA, despite convergence of scores at later follow-up^[^20^]^. Additionally, studies evaluating the VELYS and MAKO systems have reported modest reductions in complication rates and improved early safety profiles^[^17,21^]^. Supporting these findings, a large database analysis by Huang *et al* (2024) demonstrated significantly fewer 90-day revisits and a 53% reduction in knee-related readmissions associated with VELYS-assisted TKA^[^22^]^.

Taken together, these data suggest that while robotic assistance reliably enhances surgical execution, mechanical precision alone is unlikely to be sufficient to guarantee superior long-term functional outcomes for all patients. Instead, the benefits of RA-TKA may be most evident in early recovery, safety, and consistency of execution. This recognition underscores the need for more intelligent, patient-specific optimization strategies, providing a strong conceptual rationale for integrating AI to personalize alignment targets, soft-tissue balancing strategies, and outcome prediction beyond precision alone.

### The economic and logistical hurdles: cost-effectiveness and learning curve

The widespread adoption of RA-TKA is significantly tempered by economic and logistical considerations. The substantial upfront capital investment for purchasing a robotic system, coupled with ongoing maintenance costs and per-procedure disposables, presents a major barrier for many institutions. Economic analyses have shown that the cost-effectiveness of RA-TKA is highly dependent on surgical volume. A decision-analytic model by Hua *et al* (2022) concluded that RA-TKA was only cost-effective in hospitals performing more than 49 procedures annually, with an incremental cost-effectiveness ratio of $41 331 per quality-adjusted life-year at that threshold^[^27^]^. This finding, supported by other reports^[^28^]^, suggests that while robotic surgery may provide value in high-volume orthopedic centers, it may not be economically viable in lower-volume or resource-limited settings.

Another concern is the learning curve associated with new technology. Encouragingly, this appears to be a manageable challenge. A systematic review by Cacciola *et al* (2022) found that surgeons typically reached proficiency in operative time after approximately 15 cases. Critically, the study noted that implant accuracy and alignment did not exhibit a learning curve; precise positioning was achieved from the very first cases, and complication rates remained stable during the learning phase^[^29^]^. This highlights a key advantage of the technology: the robot acts as a safeguard, ensuring procedural accuracy is maintained even while the surgeon adapts to a new workflow.

### A precision tool awaiting greater intelligence

To sum up, the current body of evidence paints a clear picture of robotic platforms as exceptional instruments of precision. They reliably deliver on their promise to improve the accuracy of implant placement and reduce alignment outliers, a significant achievement over conventional techniques. However, this radiographic perfection has not yet translated into consistently superior long-term PROMs or proven to be universally cost-effective. The existing data, therefore, sets the stage for the next critical question: if mechanical precision alone is not the complete answer, what is missing? This gap highlights the need for a more intelligent approach, one that can leverage the precision of robotics but augment it with data-driven insights to personalize surgery, predict outcomes, and truly optimize results for every individual patient. This is the crucial role that AI is poised to fill.

## Integration of AI and ML across the surgical workflow

While robotic platforms provide the foundational precision for modern knee arthroplasty, their clinical impact is constrained by reliance on static planning and predefined execution parameters. Increasing attention has therefore focused on the integration of AI and ML as decision-support technologies, rather than autonomous systems, capable of augmenting surgical planning, execution, and postoperative management^[^30^]^. At present, AI does not replace surgical judgment but offers data-driven insights that may enhance consistency, personalization, and predictive capacity.

This emerging AI-enabled framework spans the entire patient journey, from risk-informed preoperative planning to intraoperative guidance and postoperative outcome prediction. Importantly, most AI applications in knee arthroplasty remain modular and task-specific rather than fully integrated, and their clinical adoption varies widely. The principal applications of AI/ML algorithms across the surgical continuum are summarized in Table 2.

**Table 2:** Applications of AI/ML algorithms across the TKA surgical workflow.

Surgical phase	Application/outcome variable	Algorithms applied	Best reported performance
Preoperative	Implant sizing and positioning (from imaging)	CNN and ANN	Accuracy >90%; AUC 0.84^[^31^]^
	Risk stratification (e.g., THA conversion)	ENPLR, SVM, and CNN	AUC up to 0.77^[^32^]^
Intraoperative	Real-time guidance and ligament balancing	Reinforcement learning and ANN	(Supports execution precision)^[^31^]^
Postoperative	Prediction of revision risk	ANN, SGB, Random Forest, and Logistic Regression	ANN AUC 0.85^[^33^]^
	Prediction of functional outcomes	SGB, Random Forest, SVM, ENPLR, and CNN	Avg. AUC 0.82; CNN Accuracy 91%^[^34^]^
	Prediction of clinical outcomes	ENPLR, LASSO, SVM, and Stacked Models	AUC 0.74–0.79^[^34,35^]^
	Prediction of healthcare utilization (LOS and cost)	ANN, Bayesian, SGB, and MLR	AUC 0.71–0.87^[^36,37^]^
	Prediction of readmission risk	Logistic regression	AUC 0.66^[^33^]^

### Preoperative intelligence: predictive and personalized planning

The surgical plan represents the blueprint for success in TKA, and AI is increasingly influencing how this blueprint is constructed. Rather than relying solely on population-based averages, ML models enable probabilistic, patient-specific planning by integrating multidimensional datasets.

One of the most significant advances is in automated planning from medical imaging. Deep learning algorithms, particularly Convolutional Neural Networks (CNNs), can analyze preoperative CT or MRI scans with remarkable accuracy^[^32^]^. These models can identify anatomical landmarks, segment osseous structures, and propose implant size and positioning parameters. Reported accuracies exceeding 90% demonstrate technical feasibility, and these tools may reduce manual planning time and inter-surgeon variability^[^31^]^. However, such outputs still require surgeon validation and are not yet universally standardized across platforms.

Beyond implant positioning, AI excels at patient-specific risk stratification. Conventional planning often relies on broad population averages, but ML models can predict an individual patient’s specific risks. Ensemble models like elastic-net penalized logistic regression and support vector machines integrate a wide array of inputs, including patient demographics, comorbidities, clinical scores, and biomechanical data, to forecast the likelihood of adverse events. For instance, these models can predict the risk of postoperative complications or even the long-term probability of a patient later requiring a total hip arthroplasty, providing crucial foresight for clinical decision-making^[^38^]^. This represents a shift from deterministic templating toward probabilistic decision support, enabling more informed shared decision-making rather than prescriptive automation.

### Intraoperative guidance: real-time adaptation and execution

A preoperative plan, regardless of its sophistication, remains inherently static. The intraoperative environment, by contrast, is dynamic and influenced by complex interactions among bone geometry, ligament tension, and implant positioning. AI applications in this phase primarily function as real-time analytical tools rather than autonomous controllers^[^39^]^.

ML algorithms are crucial for processing the stream of real-time sensor and kinematic data generated by robotic platforms. These data are used to provide dynamic feedback for implant alignment and ligament balancing. Techniques such as reinforcement learning and artificial neural networks (ANNs) can analyze this information to guide the surgeon in making sub-millimeter adjustments to bone cuts or implant rotation to achieve optimal soft-tissue tension^[^31^]^. In practice, these systems provide quantitative feedback rather than direct control, assisting surgeons in making incremental adjustments to bone resections or implant rotation.

Achieving appropriate ligamentous balance remains one of the most critical yet subjective elements of TKA. AI-driven feedback offers the potential to objectify this process, although robust clinical validation remains limited. At present, AI-enhanced intraoperative guidance improves reproducibility and may reduce inter-surgeon variability, but it does not eliminate the need for surgeon expertise and contextual judgment^[^40^]^.

### Postoperative prediction: forecasting recovery and personalizing care

The role of AI extends far beyond the operating room into the crucial postoperative phase. By analyzing surgical data and patient outcomes, ML models can forecast recovery trajectories, predict complications, and help clinicians personalize rehabilitation pathways.

AI models are highly effective in forecasting healthcare utilization and complications. ANNs have demonstrated high accuracy (AUC up to 0.71) in predicting a patient’s length of stay and associated hospital costs, allowing for better resource allocation^[^41^]^. More advanced models, such as random forest and stochastic gradient boosting, have been shown to outperform traditional regression in predicting the 90-day risk of readmission or the long-term risk of revision surgery (AUC up to 0.85)^[^33,35^]^. These models are best viewed as risk stratification tools rather than deterministic predictors, aiding clinicians in identifying patients who may benefit from closer follow-up.

Furthermore, AI contributes to tailoring rehabilitation and monitoring implant survivorship. CNNs can be applied to postoperative radiographs to automatically detect early signs of implant migration or malalignment, flagging potential issues long before they become symptomatic^[^42^]^. Other models can predict a patient’s likely functional outcome based on their preoperative characteristics and intraoperative data, helping to set realistic expectations and design personalized rehabilitation protocols that target a patient’s specific needs^[^34^]^. Such tools may help align patient expectations and personalize rehabilitation pathways, although prospective validation and integration into clinical workflows remain limited.

Taken together, these applications illustrate that AI currently enhances discrete components of the surgical pathway. The challenge, and opportunity, lies in safely integrating these tools into a coherent, clinically validated system capable of learning across the continuum of care^[^43^]^.

## The synergy of the AI-driven robotic ecosystem

The separation of robotic systems and AI in TKA is conceptual; in practice, their true value is not additive but synergistic. However, at present, this synergy is largely aspirational rather than fully realized in routine clinical practice. The convergence of robotic precision with AI’s predictive power has the potential to create a transformative paradigm: the AI-driven robotic ecosystem^[^44^]^. This concept should be viewed as an emerging, preclinical framework rather than an established clinical system.

Rather than a single deployable technology, this ecosystem represents a developmental model for future integration, in which robotic platforms serve as precision actuators and AI functions as an adaptive decision-support layer. In its idealized form, it functions as a continuous feedback loop: AI generates a data-driven plan, the robot executes it with high fidelity, the resulting surgical and clinical data are captured, and these data are then used to iteratively train and refine AI models for subsequent patients^[^45^]^. While elements of this loop exist independently, their full closed-loop integration remains limited.

**Figure 2.**
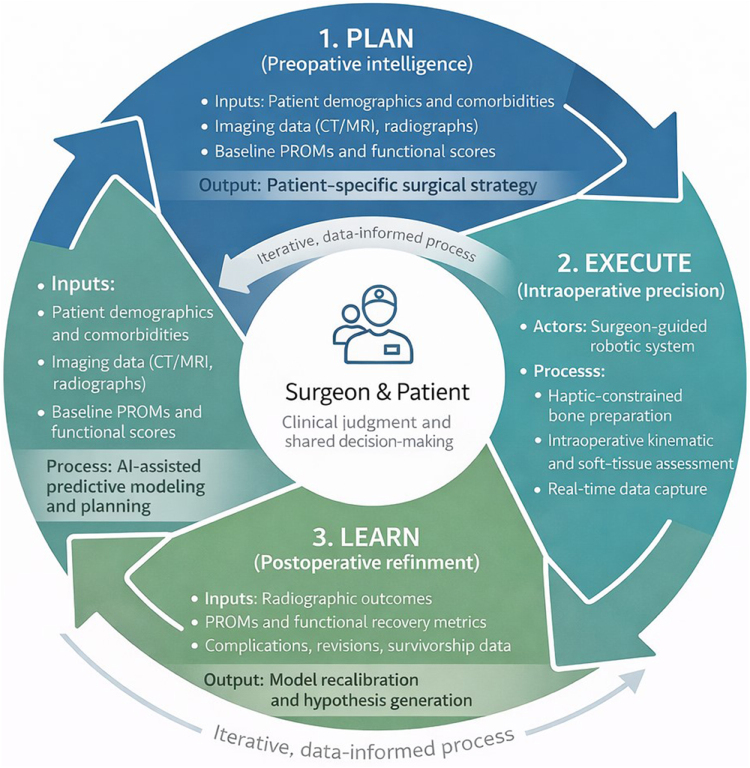
Conceptual framework of the AI-driven robotic ecosystem in total knee arthroplasty.

Note: The figure illustrates a cyclic, data-informed workflow linking preoperative AI-assisted planning, robot-enabled intraoperative execution, and postoperative data-driven model refinement. In the planning phase, patient-specific clinical, imaging, and functional data inform predictive algorithms to support individualized surgical strategies. During execution, robotic platforms enable high-fidelity implementation of the surgical plan with real-time data acquisition. Postoperatively, clinical outcomes, radiographic assessments, and PROMs are aggregated to iteratively refine predictive models. While individual components of this loop are currently implemented in isolation, full closed-loop integration remains an emerging and largely preclinical paradigm

Nonetheless, this conceptual ecosystem provides a coherent framework for addressing the persistent gap between achieving surgical accuracy and optimizing long-term, patient-specific outcomes. This dynamic process is illustrated in Figure 2.

### The feedback loop: from plan to precision and back

The engine of this ecosystem is its cyclical, data-driven workflow. In its current form, this workflow is partially implemented rather than fully autonomous. The process begins with the AI-powered preoperative blueprint, where ML algorithms analyze patient-specific imaging, demographic, and biomechanical data to generate a surgical plan intended to optimize predicted outcomes, rather than solely targeting generic mechanical alignment parameters^[^31,33^]^.

Next, this intelligent plan is translated into action through robotic precision execution. The robotic platform acts as the high-fidelity actuator of the AI’s strategy, performing bone resections and guiding implant placement with a level of accuracy and reproducibility that manual techniques cannot consistently achieve^[^16,18^]^. At present, however, the robot primarily executes predefined plans rather than dynamically modifying them based on autonomous AI decision-making. This step nonetheless ensures that the surgical plan is carried out with minimal deviation, strengthening the link between planned intent and achieved execution.

The final and most critical phase is data capture and algorithmic refinement^[^46^]^. Every aspect of the procedure, ranging from intraoperative soft-tissue balancing data to postoperative PROMs, radiographic assessments, and long-term implant survivorship, can theoretically be structured as reusable data inputs^[^47^]^. In practice, however, such datasets remain fragmented across platforms, institutions, and registries. When sufficiently large and standardized datasets are available, AI models may identify subtle patterns and correlations that are not readily apparent to human analysis, thereby improving predictive accuracy^[^48^]^. This learning capacity offers a potential solution to a major limitation of current evidence: the scarcity of long-term, high-resolution outcome data^[^8^]^.

### Implications: democratizing expertise and personalizing care

The establishment of an AI-driven robotic ecosystem has profound implications for the practice of arthroplasty. First, it promises to shift the goal from standardization to true personalization^[^40^]^. Rather than applying a single alignment philosophy to all patients, AI-enabled systems could eventually learn which combinations of implant positioning, alignment strategy, and soft-tissue balancing are associated with optimal outcomes in specific patient phenotypes^[^49^]^.

Second, this ecosystem has the potential to partially democratize surgical expertise. By aggregating data and experience from large numbers of procedures across surgeons and institutions, AI-driven guidance systems may support decision-making, particularly for less experienced surgeons^[^50^]^. Importantly, this should be viewed as augmentation rather than replacement of surgical judgment. If appropriately validated, such systems could help reduce inter-surgeon variability and flatten learning curves, contributing to more consistent outcomes across different practice settings^[^45^]^.

### Barriers to the smart future: challenges of a connected system

The realization of this vision is accompanied by substantial challenges that extend beyond those associated with standalone robotic platforms. The most significant barrier is data infrastructure and interoperability. The performance of any AI-driven ecosystem is fundamentally dependent on the quality, completeness, and standardization of input data, yet current surgical data remain highly heterogeneous across institutions and proprietary systems^[^51^]^. Overcoming issues of data heterogeneity between institutions and ensuring patient privacy are critical logistical and ethical tasks^[^48^]^.

Furthermore, the “black box” nature of many advanced AI models raises important concerns regarding clinical trust, transparency, and adoption. Surgeons may be reluctant to act on intraoperative recommendations generated by algorithms whose internal logic is not interpretable. This challenge is compounded by the risk that AI models may inherit or amplify biases present in their training data, particularly if derived predominantly from high-volume or well-resourced centers. These issues are closely linked to unresolved questions of accountability and liability. In cases of adverse outcomes following AI-assisted decision-making, determining responsibility among surgeons, institutions, and technology developers remains legally and ethically complex^[^52,53^]^.

Finally, the economic viability of a fully integrated ecosystem remains a major barrier. The cost extends beyond the initial robotic purchase to include data management infrastructure, analytical software, and specialized personnel^[^54^]^. Proving the long-term cost-effectiveness of this comprehensive system, particularly in resource-limited settings, will be essential for its widespread adoption^[^55,56^]^. Future progress will likely depend on scalable system designs, regulatory clarity, and reimbursement models that emphasize long-term value rather than procedural novelty^[^57^]^.

### Limitations

This review has several important limitations that warrant careful consideration. First, as a structured narrative review, it provides a conceptual and thematic synthesis rather than a systematic evaluation of the literature. A formal risk-of-bias assessment using standardized tools (e.g., GRADE or ROBINS-I) was not performed, and although the search strategy was comprehensive and transparent, it was not exhaustive. Consequently, selection bias cannot be fully excluded, and the relative weighting of individual studies reflects interpretive synthesis rather than quantitative hierarchy. Second, the current evidence base for RA-TKA remains methodologically heterogeneous and temporally limited. Most available studies report short- to mid-term outcomes, with relatively sparse data on long-term implant survivorship beyond 10 years. This constrains definitive conclusions regarding durability, revision risk, and long-term cost-effectiveness.

Third, a substantial proportion of the published literature, particularly studies evaluating specific robotic platforms, is industry sponsored, raising the potential for reporting and publication bias. While high-quality randomized and registry-based studies are emerging, independent long-term evaluations remain limited. Finally, although this review proposes the conceptual framework of an AI-driven robotic ecosystem, this model is largely theoretical and developmental. Fully integrated, continuously learning AI–robotic systems have not yet undergone robust prospective clinical validation, and their regulatory, ethical, and economic implications remain unresolved. Accordingly, conclusions regarding their future impact should be interpreted as hypothesis generating and forward looking, contingent upon rigorous longitudinal studies and real-world implementation data.

## Conclusion

RA-TKA has firmly established itself as a reliable means of improving surgical accuracy and reducing alignment variability. However, the evidence synthesized in this review indicates that mechanical precision alone is insufficient to consistently deliver superior long-term patient-reported outcomes, highlighting an important limitation of current robotic paradigms. This narrative review argues that the future of knee arthroplasty lies not in further refinement of precision alone but in the responsible integration of robotics with data-driven AI. AI offers the potential to augment robotic systems by enabling predictive preoperative planning, objective intraoperative decision support, and individualized postoperative outcome forecasting. When conceptually integrated, these technologies form an emerging AI-driven robotic ecosystem capable of learning from aggregated surgical and outcomes data to support more personalized and reproducible care.

Importantly, this ecosystem remains largely developmental, and its clinical impact has yet to be fully validated. Significant challenges related to data quality, algorithmic transparency, regulatory oversight, cost-effectiveness, and ethical accountability must be addressed before widespread implementation is feasible. In this evolving paradigm, the role of the surgeon remains central, with AI functioning as a decision-support tool rather than a replacement for clinical judgment. Future research should prioritize prospective clinical validation, long-term outcome assessment, and robust economic evaluation of integrated AI–robotic systems. Addressing these gaps will be essential to determine whether this synergistic approach can meaningfully bridge the gap between surgical accuracy and patient-centered outcomes and ultimately justify its adoption in routine clinical practice.

## Data Availability

No new datasets were generated or analyzed in the preparation of this manuscript.

## Research registration unique identifying number (UIN)

Not applicable, as this article is a narrative review and does not involve human participants or systematic review methodology requiring registration.
